# How Viruses Contribute to the Pathogenesis of Hemophagocytic Lymphohistiocytosis

**DOI:** 10.3389/fimmu.2017.01102

**Published:** 2017-09-07

**Authors:** Ellen Brisse, Carine H. Wouters, Graciela Andrei, Patrick Matthys

**Affiliations:** ^1^Laboratory of Immunobiology, Rega Institute, KU Leuven, Leuven, Belgium; ^2^University Hospital Gasthuisberg, Leuven, Belgium; ^3^Laboratory of Virology and Chemotherapy, Rega Institute, KU Leuven, Leuven, Belgium

**Keywords:** hemophagocytic lymphohistiocytosis, macrophage activation syndrome, pathogenesis, herpesviruses, DNA viruses, infection, immuno-evasion

## Abstract

Hemophagocytic lymphohistiocytosis (HLH) is a life-threatening, hyperinflammatory syndrome, characterized by the uncontrolled activation of macrophages and T cells, eliciting key symptoms such as persistent fever, hepatosplenomegaly, pancytopenia, hemophagocytosis, hyperferritinemia, and coagulopathy. Viral infections are frequently implicated in the onset of active HLH episodes, both in primary, genetic HLH as in the secondary, acquired form. Infections with herpesviruses such as Epstein–Barr virus and cytomegalovirus are the most common. In autoimmune diseases, a link between viral infections and autoreactive immune responses has been recognized for a considerable time. However, the mechanisms by which viruses contribute to HLH pathogenesis remain to be clarified. In this viewpoint, different factors that may come into play are discussed. Viruses, particularly larger DNA viruses such as herpesviruses, are potent modulators of the immune response. By evading immune recognition, interfering with cytokine balances and inhibiting apoptotic pathways, viruses may increase the host’s susceptibility to HLH development. In particular cases, a direct connection between the viral infection and inhibition of natural killer cell or T cell cytotoxicity was reported, indicating that viruses may create immunological deficiencies reminiscent of primary HLH.

## Introduction

Hemophagocytic lymphohistiocytosis (HLH) comprises a heterogeneous group of life-threatening, hyperinflammatory syndromes, occurring in children and adults. It is characterized by hypercytokinemia and uncontrolled activation of macrophages and T cells, eliciting key symptoms such as persistent fever, hepatosplenomegaly, pancytopenia, hemophagocytosis, hyperferritinemia, and coagulopathy (1, 2). An inherited and acquired form of HLH are distinguished, termed primary and secondary HLH. Primary HLH is caused by mutations in genes implicated in granule-mediated cytotoxicity, impairing the function of natural killer (NK) and CD8^+^ cytotoxic T lymphocytes (CTLs) (3), or can develop as a complication in X-linked lymphoproliferative disease (XLP; XLP1 and XLP2) in which mutations in *SAP* or *XIAP* confer an increased susceptibility to HLH, particularly following infection with Epstein–Barr virus (EBV) (4, 5). In contrast, no clear genetic background has been associated with secondary HLH, although accumulating evidence indicates some extent of overlap between both subtypes. An increasing number of reports suggests the involvement of hypomorphic or monoallelic mutations in cytotoxicity genes known to be associated with primary HLH, in patients with secondary HLH (6–11). In general, secondary HLH presents as a rare complication of various medical conditions, including infections, autoinflammatory and autoimmune disorders, malignancies, metabolic syndromes, and acquired immunodeficiencies (1, 12). HLH associated with rheumatologic conditions is also termed “macrophage activation syndrome” (MAS) (13).

Insights into the pathogenesis of HLH have been predominantly derived from murine models of primary HLH, in which excessive production of IFN-γ by hyperactivated CTLs has been designated as a major underlying disease mechanism (14–17). Sustained activation of CTLs was shown to be mediated by a reversal of the interleukin (IL)-2 consumption hierarchy, whereby the expression of the IL-2 receptor α chain (CD25) on CTLs surpassed the expression on regulatory T cells, resulting in a collapse of Treg cell numbers (18). IFN-γ was demonstrated to directly stimulate macrophage activation, instigating the onset of hemophagocytosis and possibly causing anemia and cytopenias in other blood cell lineages (19). These data constituted the rationale for initiating clinical trials using anti-IFN-γ antibodies in patients with severe and recurrent HLH (20). However, it should be noted that IFN-γ is not strictly necessary for the development of HLH, as is evident from HLH models in IFN-γ-deficient mice or the occurrence of HLH symptoms under IFN-γ blockade (21–24), as well as reports of HLH patients with an underlying IFN-γ-receptor deficiency (25).

Since the first reports of HLH (26), viruses have been notoriously implicated in the onset of active disease, in both primary and secondary HLH. Infections with herpesviruses are the most common, predominantly EBV and human cytomegalovirus (HCMV), but also herpes simplex virus (HSV), human herpesvirus (HHV)-6, HHV-8, and varicella zoster virus (VZV), followed by other DNA viruses like parvovirus B19 and adenoviruses (27–29). Less frequently, cases of HLH arise in RNA virus infections, including different strains of influenza virus, human immunodeficiency virus (HIV), dengue, and hepatitis C. Both primary infection and reactivation from latency have been reported to trigger HLH (27–29). Importantly, patients with HLH and concomitant viral infections were shown to carry a worse prognosis, compared to non-infected patients (30, 31). In particular, HLH associated with active EBV disease is known for its aggressive progression and poor prognosis, in which EBV viral load correlates with increased disease severity and decreased survival (27, 29, 30, 32, 33).

In autoimmune diseases, a link between viral infections and autoreactive immune responses has also been recognized for a considerable time. Viruses stimulate antigen-specific adaptive immune responses that may cross-react with self-peptides showing some degree of homology to the viral antigen, thus causing autoreactive immunopathology. An example hereof is the association between a clinical history of EBV infectious mononucleosis and development of multiple sclerosis later in life, probably due to cross-recognition of myelin autoantigens by EBV nuclear antigen-specific T cells. Alternatively, intracellular self-antigens, such as DNA, RNA, or histones, that were not expressed in the thymus, are released following virus-mediated tissue damage, possibly causing the onset of autoimmune disease (34–36).

The mechanisms by which viruses contribute to HLH remain to be clarified. Different factors may play a role. Viruses, especially large DNA viruses such as herpesviruses, are potent modulators of the immune response. An estimated 50% of the herpesvirus genome is dedicated to this cause (37). Through thousands of years of co-evolution with the human immune system, viruses have learned to manipulate inflammatory defense mechanisms to ensure their survival. By actively evading effector immune responses and distorting cytokine balances, they may increase their host’s susceptibility to HLH. In particular cases, direct interference with the cytotoxic function of T and/or NK cells was reported, indicating that viruses may create immunological deficiencies reminiscent of primary HLH. Further supporting a link between viruses and HLH pathogenesis, a recent transcriptome analysis revealed striking similarities between the expression profile of PBMCs from HLH patients and patients with acute primary EBV infection, indicating EBV was capable of eliciting uncontrolled immune responses that approximate the hyperinflammation observed in HLH (38).

This viewpoint provides a concise overview of the diverse mechanisms that could connect viruses to the onset of HLH, focusing on DNA viruses and herpesviruses in particular. Although the discussed mechanisms theoretically apply to both virus-associated primary and secondary HLH, we believe that they primarily shed light on secondary HLH pathogenesis, by illustrating the capability of different viruses to induce HLH-like symptoms and/or acquired cytotoxicity defects, in the absence of the clear genetic predisposition present in primary HLH.

## Continuous Pathogen Receptor Triggering and Bone Marrow Exhaustion

**Figure 1:**
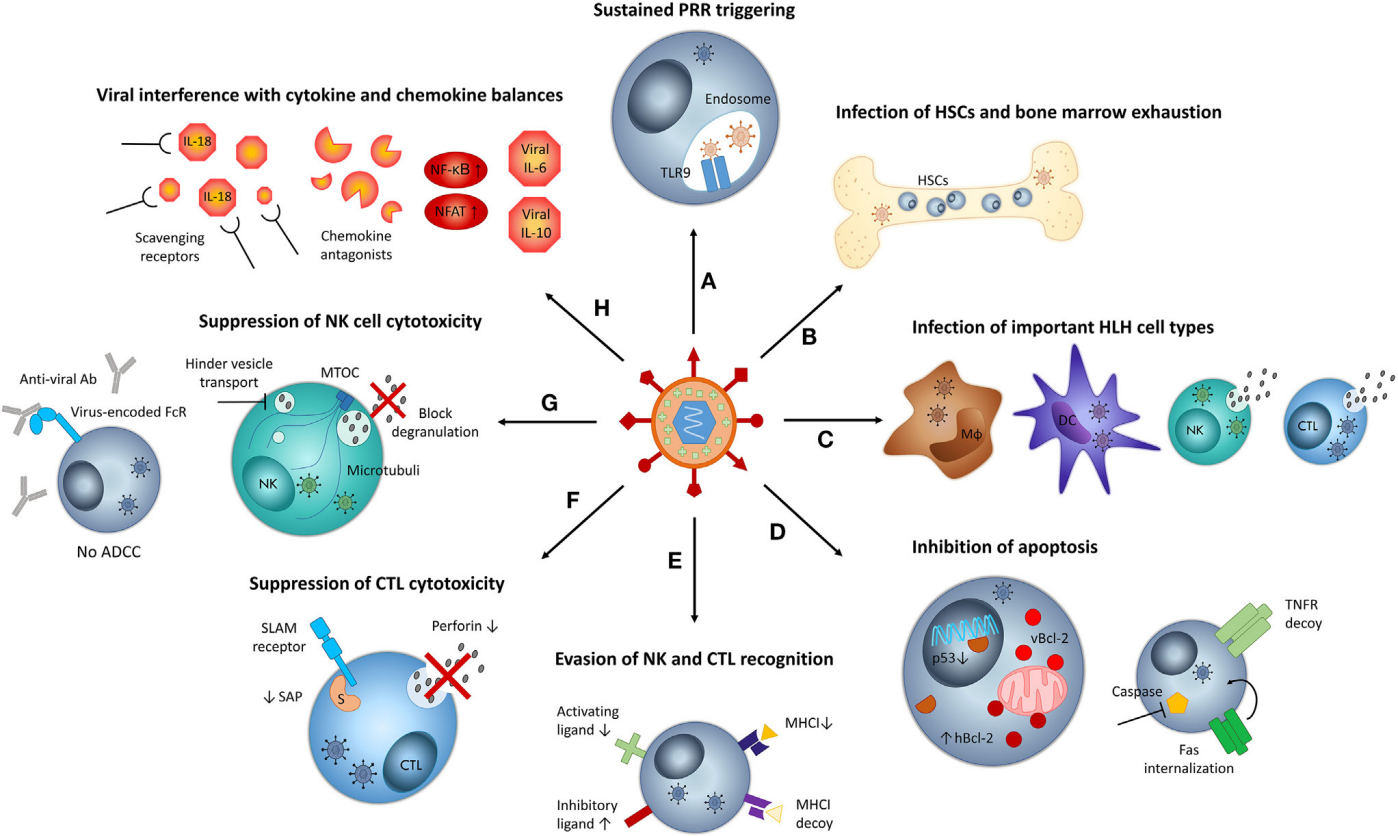
Mechanisms by which viruses may contribute to hemophagocytic lymphohistiocytosis (HLH) pathogenesis. **(A)** Sustained infections result in chronic stimulation of pattern recognition receptors (PRR), such as TLR9, mediating persistent immune cell activation. **(B)** Persistent viral infections put strain on the bone marrow, resulting in exhaustion. Some viruses may infect hematopoietic stem cells (HSCs). Both aspects can result in cytopenias and hemophagocytosis. **(C)** When viruses infect key cell types implicated in HLH pathogenesis, such as macrophages (Mϕ), dendritic cells (DCs), natural killer cells (NKs), and cytotoxic T lymphocytes (CTLs), their function may be altered and the host’s susceptibility to HLH may increase. **(D)** Some viruses inhibit apoptosis of infected cells by encoding viral Bcl-2 (vBcl-2), increasing expression of human Bcl-2 (hBcl-2), downregulating p53, encoding tumor necrosis factor (TNF) receptor (TNFR) decoys, and internalizing FAS or inhibiting the function of caspases. Inhibition of apoptosis contributes to cytokine storm development in HLH. **(E)** Several viruses circumvent recognition by CTLs *via* the downregulation of major histocompatibility complex class I (MHC-I) molecules. To avoid NK cell recognition caused by “missing self,” viruses encode MHC-I homologs, downregulate ligands of activating NK cell receptors, or upregulate ligands of inhibitory NK cell receptors, among others. **(F)** Some viruses actively suppress cytotoxic T lymphocyte (CTL) function by decreasing the levels of perforin or signaling lymphocyte activation molecule (SLAM)-associated protein (SAP, indicated by “S”). **(G)** Some viruses suppress NK cell cytotoxic function, by blocking degranulation or by encoding Fc receptors (FcRs) that capture virus-specific antibodies (Abs) and thus inhibit antibody-dependent cellular cytotoxicity (ADCC). Some viruses hinder vesicle transport along microtubules and may thus impede degranulation. **(H)** Several viruses interfere with cytokine and chemokine balances by encoding “virokines” such as viral interleukin (IL)-6 or IL-10, by stimulating nuclear factor κB (NF-κB) and nuclear factor of activated T cell (NFAT) signaling, by capturing NK cell-stimulating cytokines such as IL-18 or by expressing chemokine antagonists that impair NK cell recruitment. MTOC, microtubule-organizing center.

Following primary infection, herpesviruses like EBV and HCMV establish lifelong latency in their hosts, with sporadic reactivations. In this way, herpesviruses chronically burden the immune system with small amounts of viral antigen and require constant immune vigilance to contain the infection. By chronically triggering pattern recognition receptors (PRRs) such as toll-like receptors (TLRs), NOD-like receptors, and RIG-I-like receptors, viruses stimulate innate immunity in a sustained manner (Figure 1A) (34). Herpesviruses, carrying a double-stranded DNA genome, predominantly activate TLR9, which recognizes unmethylated CpG sequences. Interestingly, chronic and excessive TLR9 stimulation has already been linked to HLH development in two independent mouse models (39, 40), corroborating persistent virus-mediated PRR triggering as a possible causal factor in the onset of HLH.

Sustained viral infections also put strain on the bone marrow. Antiviral immune responses signal the bone marrow to provide the necessary blood cells to combat the invading pathogen, triggering stress-induced hematopoiesis. During chronic infections, this inflammatory stimulus may eventually lead to bone marrow exhaustion. Thus, many viruses provoke bone marrow pathology like pancytopenia, a characteristic feature of HLH (41). Among others, EBV, HCMV, HHV-6, VZV, hepatitis A and C virus, dengue virus, and parvovirus B19 cause cytopenias *via* the process of bone marrow exhaustion or aplastic anemia and ensuing bone marrow failure (Figure 1B) (41, 42). This bone marrow exhaustion during chronic viral infections could, together with the consumption of bone marrow cells by hemophagocytic macrophages, contributes to the development of profound cytopenias in HLH patients.

## Infection of Key Cell Types in HLH and Hematopoietic Stem Cells (HSCs)

Most herpesviruses possess the capacity to directly infect cells of the immune system. When cell types involved in HLH pathogenesis are targeted (Figure 1C), this may constitute a predisposing factor to HLH. It has been hypothesized that viral infection of cells impairs their function, as viral entry disrupts normal cellular processes and induces stress (28, 43). EBV infects B cells and nasopharyngeal epithelial cells in classical cases. However, in EBV-associated secondary HLH, it ectopically infects CTLs and, to a lesser extent, NK cells (44). This peculiar feature may not be coincidental considering the central role of these cytotoxic cells in primary HLH pathogenesis. The H1N1 influenza virus, associated with secondary HLH in severe cases, has also been described to directly infect and replicate in NK cells (45). In turn, HCMV naturally infects and establishes latency in dendritic cells (DCs) and macrophages, permitting the virus to exercise control over antigen presentation, another process implicated in primary HLH pathogenesis (46). For other viruses, cell subsets involved in HLH pathogenesis may not present the main cellular target, but can be infected occasionally. Examples include HHV-6 with a predominant CD4^+^ T cell tropism that may infect NK and CTLs (47–49), HHV-8 that infects macrophages (50), HIV that sporadically infects NK cells, HSV-1 that can infect T cells, NK cells and DCs (51, 52), and VZV that may infect T cells and DCs (53). The exceptional infection of key HLH cell types by certain HLH-associated viruses may explain why only a small percentage of infected patients develops HLH-like disease.

Infections can influence the cell’s susceptibility to apoptosis and/or activation-induced cell death and cause immortalization (54, 55). HIV-infected NK cells showed reduced viability (51), and severe H1N1 cases displayed transient NK cell deficiency (56). The infection of DCs by different influenza virus strains has additionally been linked to the development of lymphopenia, because these DCs migrate to the thymus to infect and destruct thymic cells (56). On the other hand, lymphotropic viruses like EBV, HHV-6, and HHV-8 often cause lymphoproliferation (57), and ectopic infection of CTLs in EBV-associated HLH can result in the immortalization and persistence of cytokine-producing CTLs (28). Viral infection may thus contribute to two distinct features of HLH: uncontrolled lymphoproliferation and/or lymphopenia.

In addition to infecting immune effector cells, several viruses, including herpesviruses and hepatitis C virus, infect HSCs, immune progenitor cells, or supportive stromal cells in the bone marrow (Figure 1B). This impairs the survival and proliferation of HSC, suppresses hematopoiesis, and may also result in the recruitment of cytotoxic responses to the bone marrow. For example, parvovirus B19 has a tropism for erythroid progenitor cells and induces anemia by inhibiting their cell division. In addition, the infected progenitor cells will become targeted by antiviral, cytokine-secreting CTLs, or by activated, hemophagocytic macrophages, causing further cell death and anemia. Recruitment of inflammatory cells to the bone marrow also causes collateral damage to cell types other than the targeted ones, contributing to the development of cytopenias in other cell lineages (41).

## Immune Evasion

Larger DNA viruses, like herpesviruses, utilize methods to actively evade immune recognition and elimination, methods which may be encoded by genes bearing sequence homology to human genes. These viral immunoregulatory proteins are often called evasins. The presence of host-derived sequences in the genome of different DNA viruses suggests that during years of co-evolution, viruses have efficiently “pirated” human genes to modify to their own benefit. To link viral infections with HLH onset, we will specifically discuss viral evasion strategies that allow the virus to resist apoptosis, inhibit cytotoxicity, and interfere with cytokine and chemokine balances.

### Resisting Apoptosis

Viruses encode a large variety of antiapoptotic proteins that inhibit or delay apoptosis of infected cells (Figure 1D). Without successful induction of apoptosis, cytotoxic effector cells fail to disengage from target cells, resulting in prolonged synapse duration, stimulating repetitive Ca^2+^-signaling and cytokine hypersecretion by the cytotoxic cells. Viral inhibition of apoptosis may thus contribute to cytokine storm formation in HLH (58). Antiapoptotic viral proteins can interfere with the extrinsic or intrinsic pathway of apoptosis. The former is initiated by binding of tumor necrosis factor (TNF), Fas ligand, or TRAIL to death receptors like Fas and the TNF receptor, while the latter is regulated by Bcl-2 proteins governing mitochondrial membrane permeability. Both pathways ultimately converge in the activation of different caspases that induce cell death (43).

Regarding the extrinsic pathway, HCMV encodes a decoy TNF receptor and EBV deliberately recruits components of the TNF receptor to avoid TNF-mediated apoptosis (37, 55). Adenoviruses express multimeric complexes that internalize Fas from the cellular surface, predestining it for lysosomal degradation, to prevent Fas-dependent cell death (55). Targeting the intrinsic pathway, EBV encodes two Bcl-2 homologs and additionally upregulates the expression of host Bcl-2 in infected cells. HHV-8 and adenoviruses also express Bcl-2 homologs to prevent premature death of infected cells that would limit virion production (43, 55, 59). Several viruses, including HCMV and adenoviruses, also interfere in an indirect manner with Bcl-2, by blocking or downregulating p53, a transcription factor controlling the expression of Bcl-2 proteins (43, 55). Finally, HCMV encodes a viral mitochondrial inhibitor of apoptosis, which neutralizes proapoptotic signals (43).

Downstream of both pathways, viruses such as HHV-8, HCMV, and adenoviruses have developed ways to inhibit the activation and function of caspases, including caspase 8, effectively averting cell death (37, 55, 60, 61).

### Interfering with CTL and NK Cell Cytotoxic Function

Cytotoxic T lymphocytes and NK cells are essential cytotoxic cells mediating clearance of intracellular infections. To avoid CTL recognition, viruses have developed a multitude of mechanisms to downregulate the expression and antigen presentation of major histocompatibility complex class I (MHC-I) on infected cells, thus escaping antigen-specific CTL-mediated lysis (Figure 1E). However, according to the “missing self”-theory, the lack of MHC-I molecules at the cell surface predisposes infected cells to NK cell lysis, due to insufficient engagement of inhibitory receptors. To counter this, many viruses additionally encode NK cell decoys, which mimic MHC-I molecules. Another strategy includes virus-mediated downregulation or posttranslational modification of ligands for activating NK cell receptors (Figure 1E). Viruses also interfere indirectly with NK cell function by targeting chemokines and cytokines necessary for their recruitment and activation. Among others, viruses encode chemokine antagonists that hinder NK cell trafficking or scavenging proteins that capture NK cell-stimulating cytokines like IL-18 (Figure 1H). Taken together, by stimulating inhibitory receptors, inhibiting activating receptors, and blocking cytokine/chemokine signals, viruses effectively impair NK cell cytotoxicity (51, 62). Although these CTL- and NK cell-evading mechanisms are crucial for the survival of infected cells, we will not discuss them into further detail. Instead, we will focus on viral processes with a direct effect on the intrinsic cytotoxic function of CTLs and NK cells, linking viral infections to acquired cytotoxicity defects and susceptibility to secondary HLH.

A well-known example is the latent membrane protein 1 (LMP-1) of EBV that specifically inhibits the expression of signaling lymphocyte activation molecule-associated protein (SAP) in CTLs, generating an immunological defect reminiscent of that present in XLP1-associated primary HLH (63). Defective SAP expression specifically impairs the cytolytic response of CTLs to B cell-mediated antigen presentation, resulting in a failure to control EBV infection (64). The H5N1 influenza virus can dramatically reduce perforin expression in CTLs *via* direct stimulation with the hemagglutinin protein H5 (Figure 1F) (65). This strategy not only impairs CTL cytotoxic function but also inhibits removal of H5-presenting DCs, thus sustaining persistent T cell activation, a phenomenon recently linked to primary HLH (46, 65). In addition, H1N1 influenza has been described to induce apoptosis of infected NK cells, decreasing their numbers and overall cytotoxicity (45, 56). In line with the data reported for CTLs, direct contact with influenza virions or free hemagglutinin also inhibited NK cell granule exocytosis and thus cytotoxic activity (Figure 1G) (66). Similarly, direct contact with the E2 envelope protein of hepatitis C also inhibited NK cell cytotoxicity, proliferation, and cytokine production (51). Like influenza virus, HHV-6 can also productively infect and lyse NK cells to evade immune recognition (48).

Another strategy, employed by HSV-1, HSV-2, VZV, and possibly HCMV, involves the expression of Fc receptors on the surface of produced virions and infected cells, which bind virus-targeting antibodies at the Fc region to avoid antibody-dependent cellular cytotoxicity by NK cells (37, 55). Viral glycoproteins from rotavirus can bind to microtubules, hindering the movement of secretory vesicles along the cytoskeleton and probably hampering granule exocytosis in this way (Figure 1G) (67).

As NK cells are essential factors controlling CTL hyperactivation and immunopathology in primary HLH (68), maintaining a sufficiently large pool of NK cells is essential for both viral control and HLH resistance. A decline in NK cell numbers has been reported in patients with HSV-1, HIV, hepatitis B, and parvovirus B19 (42, 69), potentially favoring HLH development.

### Molecular Mimicry Targeting Cytokines and Signaling Pathways

One of the most striking aspects of viral immunoevasion is the usage of molecular mimicry. Molecular mimics are evasins that imitate components of the host’s immune response to modulate the inflammatory process toward the benefit of the virus. Several DNA viruses encode viral cytokines and chemokines (virokines), soluble cytokine and chemokine receptors (viroceptors), binding proteins, and cellular growth factors. These homologs serve different purposes. Counterintuitively, not only anti-inflammatory effects are achieved. Most viral mimics indeed neutralize pro-inflammatory cytokine activity, but some enhance the activation of certain signaling pathways to skew the immune response and interfere with Th1/Th2 and M1/M2 balances. Viral growth factors and chemokines are secreted to stimulate specific cell proliferation and recruitment to increase the virus-susceptible pool and facilitate virus dissemination. These ingenious methods may disturb immune homeostasis, disrupt cytokine balances, and even contribute to the cytokine storm (37, 61, 70). Viral cytokine homologs are held partially responsible for the pathology caused by DNA virus infections (70). A famous example is the production of viral IL-10 by EBV and HCMV (Figure 1H). Although the sequence similarity with human IL-10 and receptor affinity differ greatly between both homologs, they are both capable of suppressing virus-specific CTLs and reducing NK cell cytotoxicity (37, 43, 51, 71).

HHV-8 secretes a viral homolog of IL-6, which activates Janus kinase (JAK)-signal transducer and activator of transcription signaling in a similar way as human IL-6, but is able to act upon a wider diversity of cell types (Figure 1H) (55, 60, 72). Interestingly, JAK inhibitors like ruxolitinib were recently proposed as potential therapy in mouse models of primary and secondary HLH (73, 74). Moreover, transgenic expression of viral IL-6 in mice led to the development of splenomegaly and lymphadenopathy and increased endogenous expression of IL-6 (75), somewhat reminiscent of the IL-6-transgenic mouse model of MAS (76). In this model, constitutively high levels of IL-6 were associated with increased susceptibility to HLH and could directly inhibit NK cell cytotoxicity (77). In HHV-8 infections, it appears that viral and human IL-6 together contribute to disease severity (72).

Not only viral cytokines influence the immune response but also chemokine homologs are employed. HCMV, HHV–6, HHV-8, and HIV encode chemokine agonists responsible for chemoattraction of neutrophils or monocytes, promoting the establishment of viral latency or sustaining viral replication (37). HHV-8 also secretes a viral chemokine homolog that prevents migration of naive NK cells to the infection site (Figure 1H) (43).

In addition to mimicry, some viruses choose to selectively induce certain cytokine or chemokine responses. For instance, during later phases of infection, EBV and HHV-8 actively trigger nuclear factor κB and nuclear factor of activated T cells signaling and thus stimulate the host’s cytokine secretion (Figure 1H) (43, 55, 60, 78). Hepatitis C virus induces anti-inflammatory cytokine production, such as IL-10 and transforming growth factor β, to suppress immune activation and impair NK cell function (79). Similarly, HIV specifically stimulates host IL-10 production by regulatory B cells to inhibit CTL responses (80).

## Discussion and Conclusion

In conclusion, viruses can disrupt the immune response through a variety of mechanisms, summarized in Figure 1. Each of these strategies may appear innocent when occurring during a controlled infection. However, they become particularly relevant when the virus manages to proliferate to exceptionally high titers, surpassing a threshold of viral load beyond which the immunoevasion strategies start to weigh on the proper functioning of the immune system. Interestingly, the inhibition of NK cell cytotoxicity following severe influenza virus infections was related to the viral load, suggestive of a dose-dependent effect of this immunoevasive mechanism (66). In EBV-associated HLH, surprisingly higher viral loads are detected when compared to other EBV-associated diseases like infectious mononucleosis (33, 81), indicating that EBV-encoded immunoevasion strategies may exert a more pronounced effect in EBV-HLH as well. Supporting this hypothesis, decreased perforin levels and reduced cytotoxicity were uniquely detected in infected T cells from patients with EBV-associated secondary HLH, not in infected NK cells from patients with chronic active EBV disease (82). Of course, cell-intrinsic susceptibility to specific evasion strategies may also play a role in this difference. Nonetheless, further evidence was provided by a recent viral RNA profiling study. More highly elevated levels of EBV-encoded microRNAs (miRNAs) (BamHI A rightward transcripts miRNAs, another class of immunoevasins) were reported in patients with EBV-HLH compared to patients with EBV-associated infectious mononucleosis, urging the authors to speculate a role for these miRNAs in HLH pathogenesis. Levels of one specific miRNA, the antiapoptotic BART16-1 (83), even correlated with disease activity and remission (81). In this light, virus-encoded evasins may constitute novel biomarkers to track disease severity, evaluate treatment responses, or predict progression from acute infection to virus-associated HLH. Studies examining the contribution of viral evasion strategies to HLH pathogenesis, as well as the expression profile and respective levels of different viral evasins in HLH patients, are required to corroborate the hypotheses stated above and to determine whether these molecules could have any impact as therapeutic targets in the future.

## Author Contributions

EB wrote the manuscript, GA, CW, and PM critically revised the manuscript. All authors agree to be accountable for the content of the work.

## Conflict of Interest Statement

The authors declare that the research was conducted in the absence of any commercial or financial relationships that could be construed as a potential conflict of interest.

## Abbreviations

BART, BamHI A rightward transcripts; CTL, cytotoxic T lymphocyte; DC, dendritic cell; EBV, Epstein–Barr virus; EBNA, EBV nuclear antigen; HCMV, human cytomegalovirus; HHV, human herpesvirus; HIV, human immunodeficiency virus; HLH, hemophagocytic lymphohistiocytosis; HSC, hematopoietic stem cell; HSV, herpes simplex virus; IL, interleukin; JAK, Janus kinase; LMP-1, latent membrane protein 1; MAS, macrophage activation syndrome; MHC, major histocompatibility complex; miRNA, microRNA; NK cell, natural killer cell; NFAT, nuclear factor of activated T cells; NF-κB, nuclear factor κB; PRR, pattern recognition receptor; TLR, toll-like receptor; TNF, tumor necrosis factor; SAP, SLAM-associated protein; SLAM, signaling lymphocyte activation molecule; STAT, signal transducer and activator of transcription; TGF-β, transforming growth factor β; VZV, varicella zoster virus; XLP, X-linked lymphoproliferative disease.
